# Pemigatinib combined with immunotherapy and stereotactic body radiation therapy for FGFR2 fusion-positive advanced intrahepatic cholangiocarcinoma with brain metastasis: a Case Report

**DOI:** 10.3389/fphar.2024.1509891

**Published:** 2024-12-04

**Authors:** Jiamin Guo, Lingqi Sun, Ye Chen, Ji Ma

**Affiliations:** ^1^ Department of Medical Oncology, Cancer Center and Laboratory of Molecular Targeted Therapy in Oncology, West China Hospital, Sichuan University, Chengdu, Sichuan, China; ^2^ Sleep Medicine Center, Mental Health Center, West China Hospital, Sichuan University, Chengdu, Sichuan, China

**Keywords:** intrahepatic cholangiocarcinoma, FGFR2-fusion, brain metastasis, immune checkpoint inhibitors, stereotactic body radiation therapy

## Abstract

**Background:**

FGFR2 fusions or rearrangements occur in 13%–20% of patients with intrahepatic cholangiocarcinoma (iCCA). Pemigatinib, a representative FGFR inhibitor, is commonly used for targeted therapy in such patients. Additionally, brain metastasis (BM) is extremely rare in advanced iCCA, and there is currently no standard treatment strategy for advanced iCCA patients with BM. Stereotactic body radiation therapy (SBRT) combined with immune checkpoint inhibitors (ICIs) may exhibit synergistic antitumor effects, presenting a promising approach for advanced iCCA.

**Case presentation:**

The patient, a 58-year-old male, experienced a recurrence of iCCA following surgery and chemotherapy, with multiple metastases in the liver, lungs, and brain. Genetic testing revealed FGFR2-TXLNG-fusion, and the patient was treated with pemigatinib in combination with tislelizumab and SBRT for the BM, resulting in significant tumor shrinkage. Adverse events (AEs) such as liver dysfunction, nail loss, and dry mouth were observed during treatment, which were considered to be related to pemigatinib. These AEs were significantly alleviated after dose reduction and symptomatic treatment.

**Conclusion:**

This case presented a rare occurrence of FGFR2 fusion-positive iCCA with BM, with extremely limited data on treatment options and survival outcomes in such patients. Our study was the first to report the application of the treatment strategy combining pemigatinib with ICI and SBRT in this specific case. The combined therapy proved effective and well-tolerated, providing new insights for future treatment considerations.

## 1 Introduction

Cholangiocarcinoma (CCA) is the second most common primary liver cancer after hepatocellular carcinoma (HCC), with a relatively low incidence, accounting for approximately 3% of all gastrointestinal tumors (Razumilava and Gores, 2014). Based on anatomical location, CCA can be subdivided into intrahepatic CCA (iCCA), perihilar CCA, and distal CCA (Ilyas et al., 2018). Recently, the incidence of iCCA has been increasing globally (Valle JW. et al., 2021). Surgery remains the only potentially curative option for resectable iCCA, but the postoperative recurrence rate is as high as 70%–75% (Vogel et al., 2023a). Furthermore, a significant number of patients are diagnosed at an advanced stage and are inoperable. The most common metastasis sites of iCCA include intrahepatic, lymph node, lung, and bone metastases. However, brain metastasis (BM) is extremely rare and carries a poor prognosis, with a median overall survival (OS) of only 3.7 months (Liu et al., 2022). To date, there are no established treatment guidelines for these patients.

Previously, the standard first-line treatment for advanced CCA was gemcitabine and cisplatin-based chemotherapy, but the median OS was less than 1 year (Valle J. et al., 2021). In recent years, targeted therapies, including those for fibroblast growth factor receptor 2 (FGFR2) fusions, isocitrate dehydrogenase 1 (IDH1) mutations, B-Raf proto-oncogene serine/threonine kinase (BRAF) V600E mutations, neurotrophic tyrosine receptor kinase (NTRK) fusions, and human epidermal growth factor receptor 2 (HER2) amplifications (Lamarca et al., 2022), along with immune checkpoint inhibitors (ICIs), are rapidly changing the treatment landscape for patients with advanced CCA (Mauro and Forner, 2023).

FGFR2 fusions or rearrangements are present in 13%–20% of patients with iCCA (Sohal et al., 2016). Pemigatinib, futibatinib, erdafitinib, and derazantinib are representative drugs targeting FGFR2 fusion (Vogel et al., 2023b). According to the FIGHT-202 study, the objective response rate (ORR) of patients with advanced CCA harboring FGFR2 fusions/rearrangements treated with pemigatinib was 35.5%, with three patients achieving complete response (CR) and a disease control rate (DCR) of 82%. Compared to other FGFR mutations and non-FGFR mutation cohorts, ORR, progression-free survival (PFS), and OS were significantly improved (Abou-Alfa et al., 2020). Other FGFR inhibitors have also shown high response rates in phase I/II trials, particularly in patients with FGFR2 fusions (Lamarca et al., 2020). However, these studies primarily focused on FGFR2 inhibitors as monotherapy. Previously, numerous studies suggested that radiation therapy stimulated a strong anti-tumor immune response by impacting nearly all steps of the cancer-immunity cycle, transforming typically immune-deserted “cold” tumors into “hot” tumors with rich lymphocyte infiltration, thereby enhancing the efficacy of ICI therapy (Zhang et al., 2022). In addition, targeted agents, including pemigatinib, could trigger highly immunogenic forms of cancer cell death to initiate the cancer-immunity cycle (Petroni et al., 2021). These theoretical foundations supported the clinical application of combined treatment strategies. This case report discussed a patient with FGFR2-fusion positive and BM iCCA who achieved excellent therapeutic outcomes using pemigatinib, ICI, and stereotactic body radiation therapy (SBRT) for BM. Additionally, relevant literature was reviewed to explore the application of FGFR inhibitors in CCA and the potential synergistic effects of combination therapies.

## 2 Case report

The patient is a 58-year-old Chinese male. In May 2023, a routine physical examination revealed a hepatic mass, prompting the patient to seek medical care at our hospital. A CT scan showed a mixed-density mass in the posterior segment of the right lobe of the liver, suspected to be a CCA. Tumor markers revealed CA199 > 1000 U/mL and CA125 at 163 U/mL. On 3 July 2023, the patient underwent laparoscopic exploration, complex right hepatic resection, cholecystectomy, and diaphragmatic repair. During surgery, a tumor measuring approximately 6 cm × 5 cm was found in the posterior right lobe of the liver, protruding from the liver surface, invading the diaphragm, and with palpable lymph nodes at the hepatic hilum. The postoperative pathology report revealed a tumor measuring 6.0 cm × 5.0 cm × 4.8 cm with no involvement of major vessels or bile duct thrombi. The histological type was moderately to poorly differentiated adenocarcinoma (likely of small bile duct origin). No microvascular invasion (MVI) or perineural invasion was observed. The tumor locally invaded the striated muscle tissue outside the capsule. Lymph node dissection of groups 7, 8, and nine yielded 3 nodes, group 12 yielded 2 nodes, and group 13 yielded 1 node, all negative for metastasis. Based on the eighth edition of the American Joint Committee on Cancer (AJCC) staging manual, the patient was staged as pT4N0M0, IIIB. Genetic testing revealed an FGFR2-TXLNG-fusion (12.9%), TMB-L (1.92 Muts/Mb, 20%), and MSS.

**FIGURE 1:**
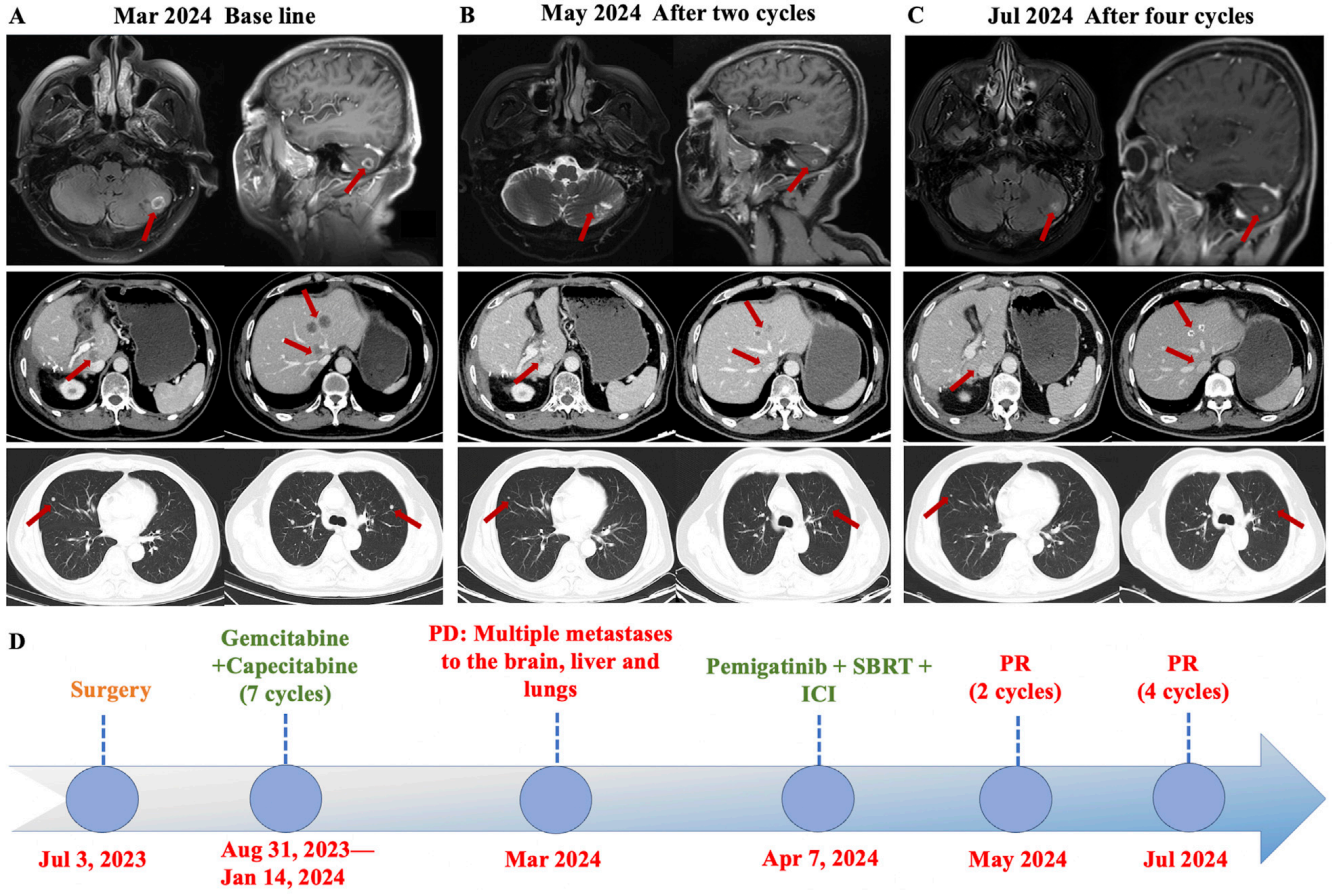
Axial CT images demonstrated changes in the patient’s brain, liver, and lung metastases at baseline and after treatment with pemigatinib combined with immune checkpoint inhibitor (ICI) and stereotactic body radiation therapy (SBRT). **(A)** The patient developed multiple brain, liver, and lungs metastases during postoperative adjuvant therapy. **(B)** The metastases in each organ were significantly reduced after two cycles of pemigatinib combined with ICI and SBRT, and the efficacy was evaluated as partial response (PR). **(C)** After four cycles of the combined therapy, the tumor shrank further and the efficacy was evaluated as sustained PR. **(D)** Timeline of the disease course and treatment. PD: Progressive Disease, PR: partially reduced, SBRT: stereotactic body radiation therapy, ICI: immune checkpoint inhibitor.

**FIGURE 2:**
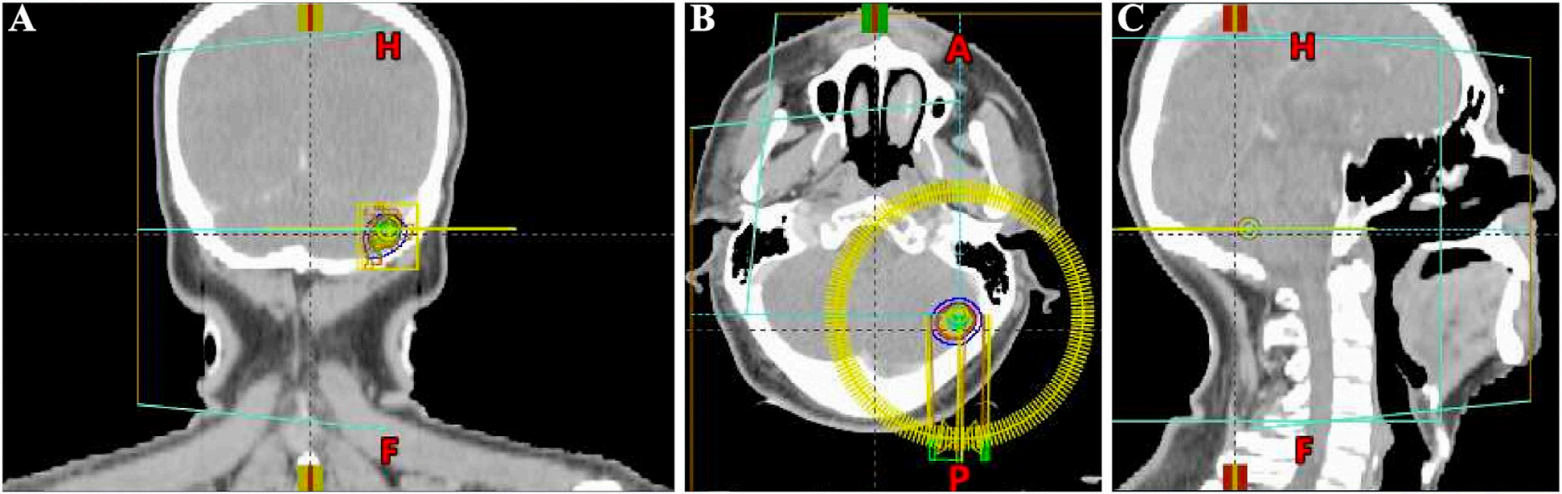
Schematic diagram of the target area for stereotactic body radiation therapy (SBRT) of brain metastasis in the patient (generated using Eclipse (v15.6, Varian Medical Systems). **(A)** Coronal head CT SBRT Target Area Diagram. **(B)** Axial head CT SBRT Target Area Diagram. **(C)** Sagittal head CT SBRT Target Area Diagram.

**FIGURE 3:**
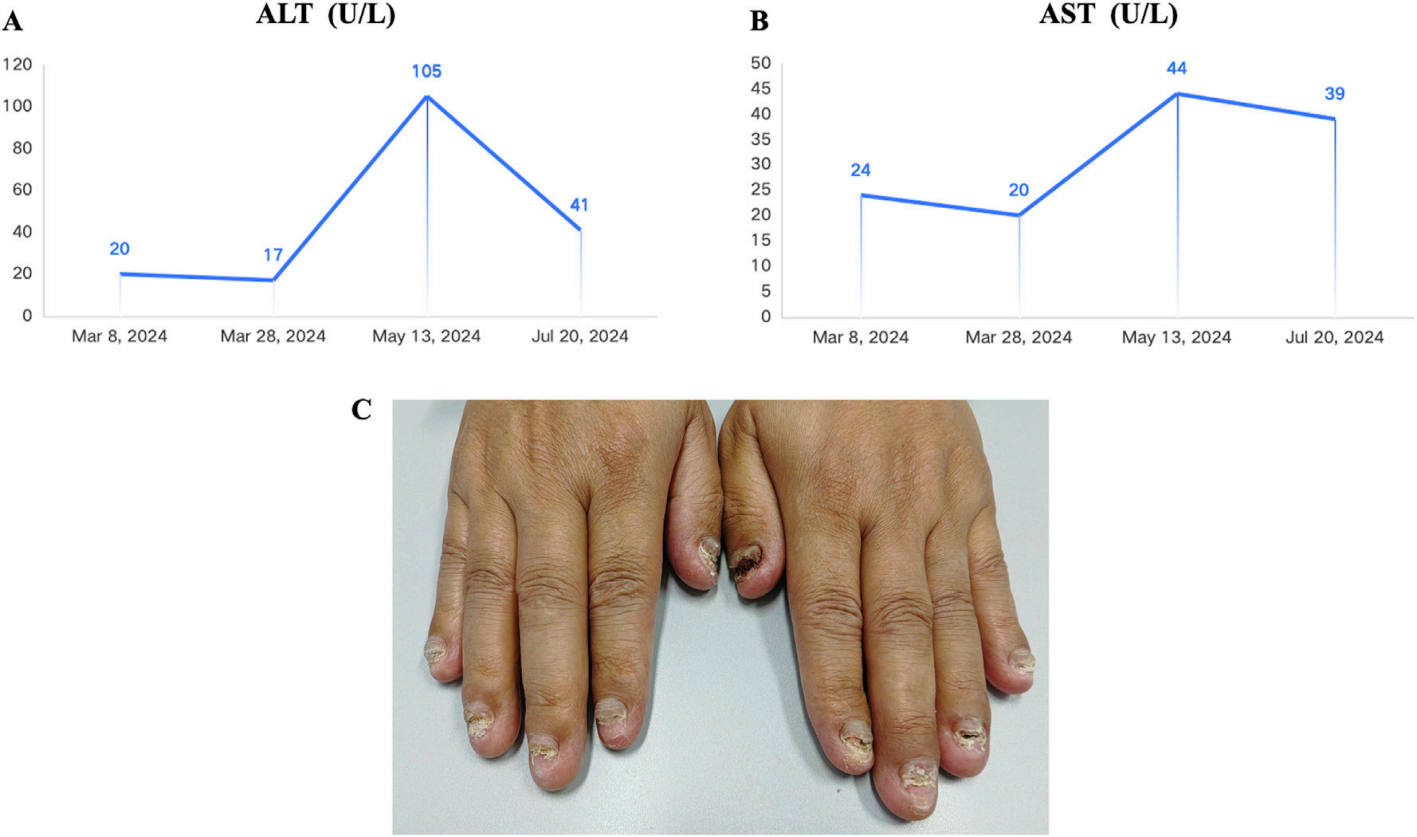
Adverse events observed in the patient during treatment with pemigatinib combined with immune checkpoint inhibitor (ICI) and stereotactic body radiation therapy (SBRT). **(A)** Alanine Aminotransferase (ALT) reached a maximum of 44 U/L during the combined treatment (Common Terminology Criteria for Adverse Events, version 5.0 (CTCAE 5.0). Grade 1). **(B)** AST (Aspartate Aminotransferase) reached a maximum of 105 U/L during the combined treatment (Grade 1). **(C)** The patient experienced nail loss during the combined treatment (Grade 1).

Considering the advanced local stage of the disease, the patient was scheduled for adjuvant chemotherapy. From 31 August 2023, to 14 January 2024, the patient underwent seven cycles of chemotherapy, receiving intravenous gemcitabine 1,600 mg (days 1 and 8) and oral capecitabine 2,000 mg (days 1–14) in 21-day cycles. After four cycles (November 2023), follow-up showed no significant tumor recurrence. After seven cycles (March 2024), imaging revealed multiple metastases in the liver, both lungs and the left cerebellar hemisphere (Figure 1). On 7 April 2024, the treatment regimen was changed to oral pemigatinib (13.5 mg daily, days 1–14) combined with tislelizumab (200 mg) every 21 days, alongside SBRT (40 Gy/5 fractions) for the cerebellar lesion. The patient’s radiotherapy plan was generated using Eclipse (v15.6, Varian Medical System) (Figure 2). After two cycles (May 2024), imaging showed that the liver, lung, and cerebellar lesions had shrunk compared to previous scans, with a partial response (PR) in tumor evaluation. The treatment was continued, and after four cycles (July 2024), follow-up imaging continued to show PR (Figure 1). During treatment, the patient developed abnormal liver function, with aspartate aminotransferase (AST) peaking at 44 U/L and alanine aminotransferase (ALT) peaking at 105 U/L, classified as Grade 1 according to the Common Terminology Criteria for Adverse Events, version 5.0 (CTCAE 5.0). Additionally, other adverse events (AEs) such as nail shedding (Grade 1), dry mouth (Grade 2), and dry eyes (Grade 1) were observed (Figure 3), which were considered to be pemigatinib-related AEs. The AEs significantly improved after supportive care for liver protection and dose reduction of pemigatinib (first from 13.5 mg to 9 mg, then further reduced to 4.5 mg due to intolerance).

## 3 Discussion

This case report presented a rare instance of advanced FGFR2-fusion positive iCCA with multiple metastases to the liver, lungs, and brain, which was successfully managed with a combination of the FGFR inhibitor pemigatinib, ICI, and SBRT targeting the brain lesion. Given the low incidence of BM in iCCA and the promising therapeutic potential of FGFR2 as a target, this article provided a comprehensive overview of FGFR inhibitors, relevant clinical studies, and the potential benefits of combining systemic and local therapies, offering valuable clinical evidence for managing such cases.

Fibroblast growth factor (FGF) ligands and receptors (FGFR1-4) play a crucial role in cancer development by activating mitosis and mesodermal signaling pathways (Silverman et al., 2021). In biliary tract cancers (BTC), FGFR2 fusions are predominantly observed in iCCA patients (Goyal et al., 2021). FGFR inhibitors hold significant clinical potential in iCCA patients, as they effectively inhibit the FGF signaling driven by FGFR2 gene fusions or rearrangements, resulting in favorable therapeutic responses (Katoh et al., 2024). Currently, two adenosine triphosphate (ATP)-competitive reversible FGFR inhibitors, infigratinib (BGJ398) and pemigatinib, have been approved by the American Food and Drug Administration (FDA) for iCCA patients with FGFR2 fusion/rearrangement who have progressed after first-line chemotherapy (Kang, 2021; Hoy, 2020). In phase II clinical trials (CBGJ398 × 2204 and FIGHT-202), infigratinib and pemigatinib achieved ORRs of 23.1% and 35.5% in these highly selected patients, respectively (Javle et al., 2021; Abou-Alfa et al., 2020). Furthermore, futibatinib, a next-generation covalent FGFR1-4 inhibitor, demonstrated efficacy in a phase II clinical study (FOENIX-CCA2), showing an ORR of 42% in patients with FGFR2 fusion/rearrangement-positive iCCA, with median PFS and OS of 9.0 and 21.7 months, respectively (Goyal et al., 2023). Table 1 summarizes the key clinical studies on FGFR inhibitors in FGFR2 fusion/rearrangement-positive iCCA patients (Javle et al., 2021; Goyal et al., 2023; Park et al., 2024; Javle et al., 2020). These studies indicated that pemigatinib, futibatinib, erdafitinib, derazantinib, and infigratinib all showed favorable efficacy in these patients. The most common AEs reported in these trials was hyperphosphatemia, occurring in 60%–80% of cases, which was related to the FGF23/FGFR signaling axis in phosphate homeostasis (Kommalapati et al., 2021). Other common AEs included fatigue, alopecia, gastrointestinal toxicity, nail toxicity, stomatitis, and ocular toxicity. At the time of writing, the patient has achieved 5.0 months of PFS since beginning treatment with pemigatinib in combination with ICI and SBRT, comparable to the results of the FIGHT-202 study. Moreover, none of the patients included in FIGHT-202 had BM, which is an important factor in survival. Patients with BM generally have poorer prognoses than others. In addition, compared to monotherapy in clinical studies, despite our use of a combined approach involving targeted therapy, radiotherapy, and immunotherapy, only Grade 1 liver enzyme elevation, nail loss (Grade 1), dry mouth (Grade 2), and dry eyes (Grade 1) occurred during the entire treatment period, with no Grade 3 or higher AEs observed. The combination therapy did not show excessive AEs compared to monotherapy.

**TABLE 1:** The key clinical studies on FGFR inhibitors in FGFR2 fusion/rearrangement-positive iCCA patients.

Drug (clinical trial)	Target	Phase	Sample size	ORR (%)	DCR (%)	mPFS (months)	mOS (months)	AEs	≥Grade 3 AEs (%)	References
Futibatinib (FOENIX-CCA2)	FGFR1–4	II	103	42	83	9.0	21.7	Hyperphosphatemia (85%), alopecia (33%), dry mouth (30%), diarrhea (28%), dry skin (27%), and fatigue (25%)	49	Goyal et al. (2023)
Pemigatinib (FIGHT-202)	FGFR1–3	II	107	35.5	82	6.9	21.1	Hyperphosphatemia (60%), alopecia (46%), diarrhea (34%), fatigue (31%), and dysgeusia (38%)	64	Abou-Alfa et al. (2020)
Erdafitinib	FGFR1–4	IIa	22	40.9	81.8	5.6	25.8	Hyperphosphatemia (85.7%), dry mouth (51.4%), stomatitis (48.6%), alanine aminotransferase increased (45.7%), aspartate aminotransferase increased (42.9%), diarrhea (37.1%)	62.9	Park et al. (2024)
Infigratinib (CBGJ398 × 2204)	FGFR1–3	II	108	23.1	84.3	7.3	12.2	Hyperphosphatemia (76.9%), stomatitis (54.6%), fatigue (39.8%), alopecia (38%), dry eyes (34.3%)	32.4	Javle et al. (2021)
Derazantinib (FIDES-01)	FGFR1–3	II	103	21.4	75.7	8.0	15.9	Hyperphosphatemia (73%), dry mouth and nausea (44.8%), fatigue (34.5%), vomiting (31.0%), dry eye (17.2%), conjunctivitis (13.8%), blurred vision (10.3%)	32	Javle et al. (2020)

AEs, adverse events; ORR, objective response rate; DCR, disease control rate; PFS, progression-free survival; OS, overall survival.

The occurrence of BM in iCCA is extremely rare. Xie P et al. reviewed 20 cases of iCCA with BM, reporting a median OS of 5.7 months, which was significantly shorter than that seen in other malignancies with BM (Xie et al., 2022). Currently, there is limited experience and evidence regarding the treatment of iCCA with BM. In the study mentioned above, 20 patients with iCCA BM were treated with different strategies. Four patients underwent further craniotomy, and 11 patients received systemic treatments such as whole-brain radiotherapy (WBRT), chemotherapy, targeted therapy, or immunotherapy. Two of these patients received a combination of immunotherapy and targeted therapy, achieving a survival time exceeding 16 months (Xie et al., 2022). This aligns with our findings, suggesting that the use of targeted therapy combined with immunotherapy could be a viable option for iCCA patients with BM. In other solid tumors, surgery is the most effective treatment for BM, and for patients who are not suitable for surgery, radiotherapy is a common local treatment option (Towards personalizing radiotherapy for brain, 2022). Nikitas J et al. reported that in non-small cell lung cancer (NSCLC) patients with brain oligometastatic, the use of SBRT and stereotactic radiosurgery (SRS) for brain lesions resulted in a 1-year local control rate of 80% and an OS of 12.4 months (Nikitas et al., 2019). Another study demonstrated that anti-PD-1 therapy combined with radiotherapy in melanoma patients with BM resulted in an objective intracranial response in 60% of patients, with 20% achieving CR. The median PFS and OS were 10.73 and 15.87 months, respectively, highlighting the potential benefit of combining anti-PD-1 therapy and radiotherapy in BM patients (Wu et al., 2021). Moreover, Kotecha R’s study found that patients receiving synchronous ICI and SRS treatment had better response rates and durability compared to those who received SRS followed by delayed ICI treatment in a cohort of 150 cancer patients with BM (Kotecha et al., 2019). Preclinical and clinical data supported the notion that immunotherapy and radiotherapy have synergistic antitumor effects (Deng et al., 2014; Dovedi et al., 2014). In this case, the patient presented with a single BM lesion was treated with high-dose radiation therapy (SBRT) to the localized tumor. SBRT can induce an immune-mediated systemic response, known as the “abscopal effect” (Kimura et al., 2022). When tumors are irradiated, stress or damage to tumor cells may cause the release of tumor-associated antigens (TAAs) from necrotic and apoptotic tumor cells and debris, thereby stimulating tumor-specific immune responses. In addition, irradiated tumor cells may also release damage-associated molecular patterns (DAMPs) and cytokines, which can enhance the traffic of immune cells (Ngwa et al., 2018). Therefore, the combination of SBRT and ICI in treating this patient likely enhanced tumor sensitivity to immune-mediated cell death, producing a synergistic anti-tumor effect.

Current research suggests that radiotherapy could disrupt the blood-brain barrier (BBB), increasing the permeability of targeted therapies (Sprowls et al., 2019). Li et al. demonstrated that radiotherapy downregulated claudin-5 and weakened the BBB, increasing anlotinib distribution in the central nervous system by 1.0–2.9 times when combined with radiotherapy for glioblastoma (Li et al., 2023). Another study reported that the BBB permeability to gefitinib increased with the radiation dose in NSCLC patients receiving WBRT and concurrent gefitinib (Zeng et al., 2015). In our study, pemigatinib was selected based on the patient’s genetic test results. Although current clinical trials and NCCN guidelines recommend using FGFR2 inhibitors as monotherapy, the limited BBB penetration of pemigatinib may reduce its efficacy in treating metastatic brain tumors. Radiotherapy may increase the permeability of the BBB, and given the theoretical basis of a synergistic effect between radiotherapy and immunotherapy, we decided to use a combination therapy of pemigatinib and SBRT for treating this iCCA patient with BM. Furthermore, the inhibition of the FGFR pathway by pemigatinib may enhance antitumor immunity and boost the activity of PD-1 inhibitors. Although pembrolizumab and durvalumab are recommended ICIs for advanced BTC (Oh et al., 2022; Kelley et al., 2023), tislelizumab, a domestically produced anti-PD-1 monoclonal antibody, was chosen due to its lower cost and greater accessibility. Previous reports, such as a case of a locally advanced iCCA patient, showed significant tumor shrinkage after treatment with gemcitabine combined with oxaliplatin (GEMOX), tislelizumab, and lenvatinib (Zhang and Yu, 2024). Another case series reported four advanced gallbladder cancer (GBC) patients who responded to tislelizumab and lenvatinib combined with chemotherapy (Wang et al., 2023). Therefore, tislelizumab may be a viable alternative for BTC patients requiring immunotherapy.

To our knowledge, no published studies reported the efficacy of FGFR inhibitors combined with PD-1 inhibitors and SBRT in treating FGFR2-fusion positive iCCA with BM. In this report, the patient developed multiple metastases in the liver, lungs, and brain during adjuvant therapy, 8 months after surgery. We used a combination of targeted therapy, immunotherapy, and radiotherapy. Remarkably, the patient tolerated the combination therapy well, and after two cycles of treatment, the metastatic tumors had all significantly shrunk. At the latest follow-up in September 2024, the tumors continued to show PR, with no new metastases.

Although our study demonstrated promising efficacy, it still has several limitations. First, the study lacks a control group and has the inherent bias of a single-case study. Furthermore, the rarity of iCCA patients with both FGFR2 fusion-positive and BM limits the generalizability of our findings to a broader patient population.

In conclusion, this case validated the efficacy of pemigatinib in FGFR2 fusion-positive iCCA. For managing iCCA with BM, the combination of radiotherapy, immunotherapy, and targeted therapy may yield synergistic anti-tumor effects with controllable toxicity, providing valuable clinical evidence for managing such highly specific cases. Additionally, it may be worthwhile to conduct more prospective randomized trials to further validate these potential clinical benefits.

## Data Availability

The original contributions presented in the study are included in the article/Supplementary Material, further inquiries can be directed to the corresponding author.
